# A topological data analysis based classification method for multiple measurements

**DOI:** 10.1186/s12859-020-03659-3

**Published:** 2020-07-29

**Authors:** Henri Riihimäki, Wojciech Chachólski, Jakob Theorell, Jan Hillert, Ryan Ramanujam

**Affiliations:** 1 University of Aberdeen, Aberdeen, UK; 2 KTH-The Royal Institute of Technology, Stockholm, Sweden; 3 Karolinska Institutet, Stockholm, Sweden; 4 University of Oxford, Oxford, UK

**Keywords:** Topological data analysis, Machine learning, Multiple measurement analysis

## Abstract

**Background:**

Machine learning models for repeated measurements are limited. Using topological data analysis (TDA), we present a classifier for repeated measurements which samples from the data space and builds a network graph based on the data topology. A machine learning model with cross-validation is then applied for classification. When test this on three case studies, accuracy exceeds an alternative support vector machine (SVM) voting model in most situations tested, with additional benefits such as reporting data subsets with high purity along with feature values.

**Results:**

For 100 examples of 3 different tree species, the model reached 80% classification accuracy after 30 datapoints, which was improved to 90% after increased sampling to 400 datapoints. The alternative SVM classifier achieved a maximum accuracy of 68.7%. Using data from 100 examples from each class of 6 different random point processes, the classifier achieved 96.8% accuracy, vastly outperforming the SVM. Using two outcomes in neuron spiking data, the TDA classifier was similarly accurate to the SVM in one case (both converged to 97.8% accuracy), but was outperformed in the other (relative accuracies 79.8% and 92.2%, respectively).

**Conclusions:**

This algorithm and software can be beneficial for repeated measurement data common in biological sciences, as both an accurate classifier and a feature selection tool.

## Background

Topological data analysis (TDA) is a recently emerging method for analyzing large-scale data using geometry and methods from algebraic topology. By considering geometric and topological features of multi-dimensional data arising from various distance metrics imposed on the data, complex relationships within the data can be preserved and jointly considered. This often leads to better results than using standard analytical tools.

There have been several publications in biological research fields which have utilized TDA successfully. These include modeling RNA hairpin folding [1], Type-2 diabetes (T2D) subgrouping using clinical parameters [2], and classification of breast cancer tumors based on gene expression patterns [3].

Despite this, TDA software typically allows only singular measurements. That is, data is often input using a single measurement point per sample. Frequently in biological data collection, multiple measurement points are taken per sample. This may occur during sampling over some time interval, or repeated measures which are indicative of sampling from a distribution of events for each individual or sample. In this case, current methods are insufficient to classify these data accurately, since all measurement points are not considered together and in an informative manner.

To address these issues, we have developed a TDA based algorithm suitable for repeated measurements which is made publicly available. This method is inspired by the Mapper algorithm and contains a classifier built on the Mapper graph generated. This is accomplished using internal cross-validation using multiple bootstraps, and as a result the partitioning is robust against overfitting. The end result is a set of subgroupings of the relevant classes in the data which can then be used as a starting point for further investigation into mechanisms behind data classification.

We test this method on three unique datasets. The first is a simulation of six different point processes on a unit square. The second example is data from 3D modeling of various tree species, using laser scanning methods to determine characteristics of tree branches. These branches act as multiple measurements from a single tree and are then used as an input to the model. The third data set is spiking activity of neuron communities in a reconstructed brain network. Neuron communities allow to make multiple measurements from a single network. We demonstrate the accuracy of this method as compared to a support vector machine (SVM) based classifier as well as determining how the accuracy changes over sampling rates, when the data available may be too large to run in total.

This paper adds to bioinformatics methods by extending TDA to classification problems within various fields, particularly for repeated measurements data for which method developing is lacking, as well as by integrating machine learning to Mapper graphs. Additionally, the sampling method used adds to the utility when large amounts of data are gathered, and classification of subsets is required. This work, and its related software, allow a user to create classifications based on large-scale data with repeated measurements, and also can report important criterion of nodes of interest, i.e. features in common with those samples which geometrically partition based on class membership, which can be used to interrogate biological processes which may be relevant for the classification and further analyses.

The paper takes the following form: first, we make a case for the use of topological data analysis while introducing the Mapper algorithm. Then, we introduce enhancements made to the Mapper algorithm to enable multiple measurements, sampling, data aggregation and classification using machine learning. We then describe three datasets used for testing purposes, each of which is indicative of a different data structure. Next, we present the results of these tests and comparisons between the methods tested. Finally, we discuss the relevance of these findings and present conclusions.

## Case for topological data analysis

### Topology of finite point sets

Topological data analysis (TDA) is not about fitting known mathematical shapes studied in topology to datapoints, but rather aims at extracting features of data based on geometry and topology encoded in the distribution of datapoints [4, 5]. Connections between datapoints correspond to relationships in the data and topological methods give insight into this relational structure. Mathematically, topology studies geometric structures only up to qualitative features, and essentially topological methods are robust against small perturbations. This is highly desirable with biological data. It allows two datasets to be close even under inherent biological variability and measurement noise. Stable results have been a focus of the theoretical development of TDA from early on [6].

A **metric** on a set *M* is a function *d*:*M*×*M*→[0,*∞*), where [0,*∞*) denotes the set of non-negative real numbers. It is required that, for any points *x*,*y*,*z* in *M*,*d*(*x*,*y*)=*d*(*y*,*x*),*d*(*x*,*x*)=0, and *d*(*x*,*z*)≤*d*(*x*,*y*)+*d*(*y*,*z*). A metric on *M* thus provides a way to measure distances between points of *M* and we refer to the pair (*M*,*d*) as a **metric space**. In TDA the initial input is given by a dataset with a chosen metric on its points. We refer to such inputs also as **point clouds**.

**Fig. 1:**
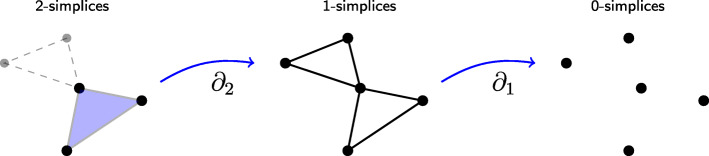
Five datapoints in two-dimensional space and the associated simplicial complex. The full simplicial complex is the purple 2-simplex and the three connected 1-simplices drawn here dashed. The boundary matrices are *∂*_1_ and *∂*_2_. Homology of this complex in degree 0 is 1 saying that there is one connected component. Homology in degree 1 is 1 indicating the loop created by the dashed 1-simplices

When a point cloud is distributed unevenly, geometric structures called **simplicial complexes** derived from the data can yield important information about dataset’s structure. Given a dataset *M*, a simplicial complex on *M* is a collection of non-empty subsets of *M*, called **simplices**, such that any subset of a simplex is also a simplex. For example, when (*M*,*d*) is a metric space, for any positive real number *r*, the collection consisting of subsets $\{x_{0},\dots,x_{k}\}\subset M$, for which *d*(*x*_*i*_,*x*_*j*_)≤*r* for every *i*,*j* in $ \{0,\dots,k\}$, is a simplicial complex called the **Vietoris-Rips** complex at scale *r*. Two datapoints at most distance *r* apart create a simplex, which can be geometrically described as an edge. These Vietoris-Rips complexes encode geometric features of the data such as connected components, or clusters, and holes. Detecting holes in data has gathered interest for example in database community [7]. **Homology** is an algebraic method to measure the amount of geometric features of different degrees. Homology in degree zero counts the number of clusters, homology in degree 1 counts the number of 1-dimensional loops, etc. Computing homology is effectively matrix computations with so called boundary matrices that contain information on how different simplices are connected to each other. See Fig. 1 for an example of a simplicial complex and its homology. An early success of TDA using homological methods came in [8] where it was discovered that the space of patches of natural images conforms to a well-known geometric object. Since then, TDA has enjoyed a multitude of applications in various areas of science, for example in [9] for finding differences in the brain arteries between ages and sexes. For a more comprehensive list of references we refer to [5].

### Mapper construction

**Fig. 2:**
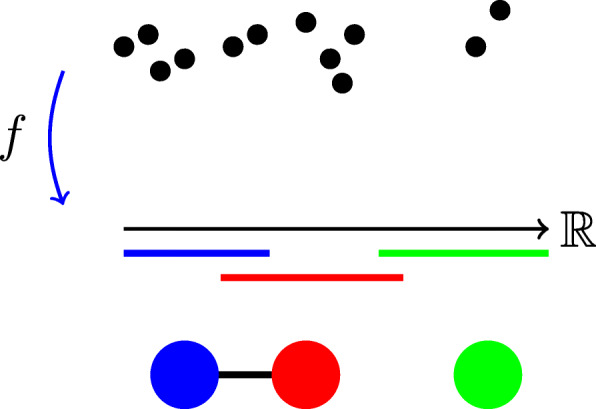
Schematics of the Mapper construction. Filter function *f* maps datapoints to intervals of real numbers. Those points mapping to the same interval constitute a cluster under the filtering by *f* and corresponding nodes in the Mapper graph. This is indicated, respectively, by blue, red and green intervals and corresponding blue, red and green nodes below. When two intervals overlap and they have datapoints in common, the corresponding nodes in the Mapper graph are connected by an edge. This happens for blue and red nodes but the datapoints in the green node are an isolated cluster

Another early advance came in [3] where a TDA algorithm called **Mapper** was used to find a new subgroup of breast cancer with an excellent survival prognosis. Figure 2 provides an easy visual complement to the explanation of the Mapper algorithm below. Mapper models **data as a graph** by refining standard clustering algorithms with topological ideas. Namely, global clustering of the data may be inefficient, especially when the data’s distance metric is not Euclidean. Instead, data is partitioned according to some intervals of real numbers $\mathbb {R}$. These intervals are created by using a **filter function***f*, meaning a function on data under which each point has exactly one value on some interval. Then, **local clustering** is achieved based on those datapoints which map to the same interval. The clusters make nodes of the Mapper graph. Intervals are overlapping by some predefined amount. Clusters with non-empty intersection of points mapping to the overlap of two adjacent intervals are then joined by an edge in the graph. If three or more clusters of points have non-empty intersection mapping to the same overlap of intervals, 2- and higher simplices can become present in the Mapper graph. The Mapper construction thus creates a simplicial complex of clusters representing the structure of data under the chosen filtering function. This modification to standard clustering gives more insight into the global structure of data through simplicial constructions as explained above. There are publicly available mapper versions, such as "Python Mapper", which can be used to analyze data in this fashion [10].

## Enhanced mapper for multiple measurements

This paper builds upon the foundations of the previous section. We have augmented Mapper by integrating a sampling procedure for the data, as well as adding a machine learning classifier which reports the unbiased accuracy of the underlying model. Important nodes of interest in the Mapper graph can be detected, which may yield important information about the data space, and relationships to the main outcome. We supplement our method shown in the pseudocode Algorithm 1 with a detailed explanation in this section.


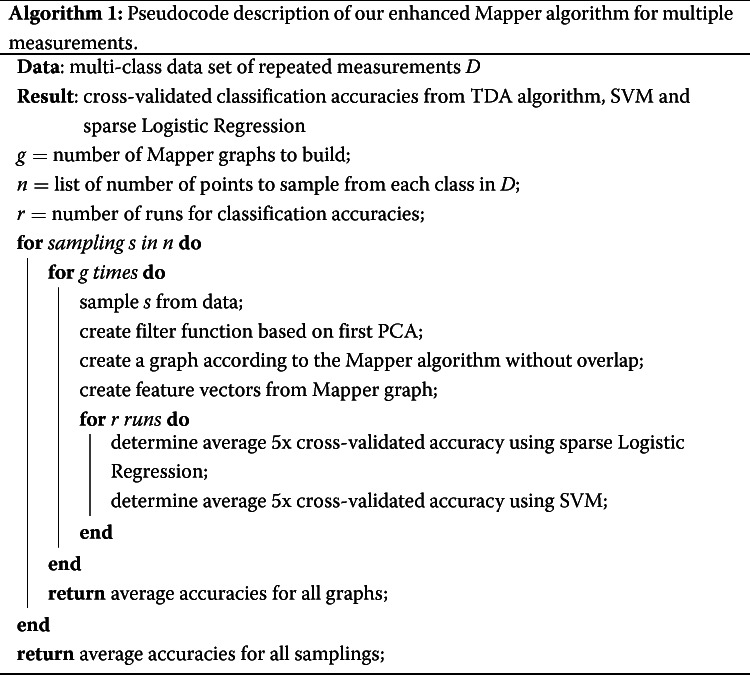


The methodology used in this manuscript is an extension of published work in the TDA field combined with a machine learning approach. Due to this algorithm being created for repeated measurements, it is important to note that the term "sample" refers to one individual or one particular example of, e.g., a tree species, which itself contains many repeated measurement points. Each of these measurements is referred to as a "datapoint".

The algorithm presented here begins by randomly sampling each sample using some number of datapoints, less than or equal to the number of datapoints of the smallest sample included. Thereafter a Mapper graph is constructed, with nodes and edges representing small clusters of datapoints and connections between the clusters as described above. Within the intervals, local clustering of datapoints is conducted and guided by standard methods. The choice of linkage method here can be changed by the user. For purposes of our algorithm edges are not necessary, and only the nodes’ contents themselves are used for analyses.

The next step of the algorithm is to add a machine learning on top of the graph structure. Nodes contain a number of datapoints from each of the samples based on data geometry detected by the chosen filtering function. This node information can be summarized in an *n* by *m* matrix where *n* is the number of samples and *m* is the number of nodes in the graph, and entries are the number of datapoints in a given node that come from the sample corresponding to *n*. These are then fed into a classifier, in this case a sparse logistic regression model was used for both binary and multi-class outcomes using the sklearn module SVC [11]. After an unbiased classification accuracy is obtained, the last step is to rerun the entire data set, constructing a Mapper graph from which feature selection can then occur based on the resultant classifier.

In order to avoid overfitting at any step, careful measures are needed. First, since this method samples from some data space, multiple samplings are conducted and the results are averaged to more accurately represent the sample distribution. Next, cross-validation is conducted, as is running multiple classifiers per graph in order to find the average results so that a particular data partitioning does not result in an over- or under-estimation of the classification accuracy.

The general procedure we used was to first determine the sampling rates to use for each data set. The sampling rates were chosen from the list [10, 20, 30, 40, 50, 60, 70, 80, 90, 100, 150, 200, 400, 500], using a maximum of 100 points for the point processes data, 400 points for the tree species, and 500 points for the neuron spiking data. For each sampling rate in the point process and neuron datasets, 10 runs were conducted of the entire procedure, and the classification accuracy was averaged for these runs. For tree species, this was increased to 100 runs due to larger variability in the results. Within each run, a graph was built upon which a cross-validated classifier could be built. The logistic regression model was created using 3-fold cross-validation, resulting in an out-of-sample prediction on each sample in the dataset. The procedure of building a classifier was repeated for an alternative model, namely an SVM model with the optimal kernel determined via testing. This kernel was linear for the tree data and radial basis function (RBF) for the point processes and neuron spiking data. For alternative accuracy, the model was built on the sampled data as opposed to the Mapper graph to provide the strongest possible alternative model. This also employed 3-fold cross-validation at the sample level, where a sample’s out-of-sample prediction was based on the majority vote of its datapoints in the training SVM. The alternative models were also constructed 10 and 100 times to account for variability on cross-validation sample assignment.

Lastly, a single model to indicate feature importance was conducted on tree species data using the graphing procedure and 400 sample points. Thereafter, information regarding the node size, average feature values for this node, and node purity were generated. Node purity is described as the proportion of datapoints in the node which belong to the largest class, such that the minimum can be 1/classes, and the maximum is 1. The average feature values for this node were determined by calculating the arithmetic mean of each feature for data in the node, providing a comparative mechanism to examine differences between nodes. This provides information regarding how values influence the outcome in a more complex manner than obtained with classical methods. The results are shown in Table 1 and we elaborate on this below in “Results” section.

**Table 1:** Top nodes by numbers of datapoints included, and average node feature values, of the tree species data with 400 datapoints from each tree sample

Node number	Datapoints	Purity	Branch order	Branch length	Branch height	Branch angle
17	23479	0.405	2.570	0.612	14.202	1.532
18	22887	0.604	2.627	0.655	17.436	1.511
14	22668	0.581	2.481	0.543	11.141	1.505
22	17634	0.766	2.710	0.650	20.322	1.494
10	13763	0.634	2.264	0.559	8.145	1.467
7	4481	0.536	1.641	0.758	5.076	1.393
6	3535	0.778	2.956	0.392	4.864	1.485
26	2614	0.600	1.947	0.860	23.349	1.453
2	2317	0.556	1.634	0.669	2.129	1.237
25	1821	0.784	3.484	0.439	23.041	1.543
9	1523	0.724	4.292	0.298	7.843	1.520
3	839	0.770	2.771	0.390	2.594	1.851
12	781	0.647	4.702	0.289	11.031	1.933

^1^Purity is defined as the highest proportion of datapoints in the node that come from a single class.

^2^Branch order is the level of branching: 0 for trunk, 1 for branches originating from trunk etc.

^3^Branch length is the branch length in meters.

^4^Branch height is the height of branch’s starting point above ground in meters.

^5^Branch angle is defined as the angle between branch’s direction at its starting point and upward z-direction in radians

## Datasets employed

Three datasets were employed for this study. The first was a simulation of six different point processes on the unit square. The second was a collection of branch data obtained from laser scanning of botanical trees. The third dataset we investigated was neuronal spiketrain data obtained from the Blue Brain reconstruction of a rat somatosensory cortex.

### Point processes

**Fig. 3:**
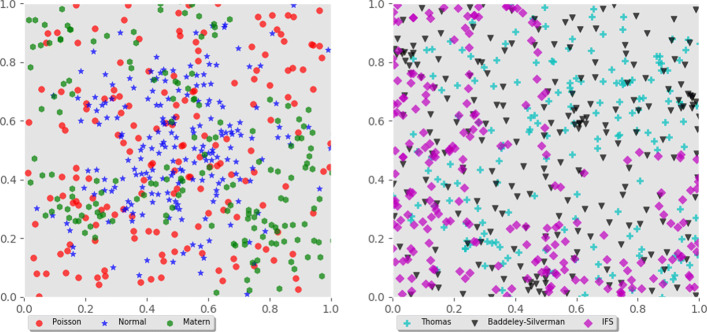
Example simulations of six different point processes used in the study

Point process refers to a configuration of points in a spatial domain according to some probability distribution. They are used to model, for example, the location of infection centers in epidemiology and spike patterns of neurons in computational neuroscience. Point processes have gathered interest in TDA community as case studies, see for example [12–14]. Let *X*∼*PD*(*k*) denote that random variable *X* follows probability distribution *PD* with parameter *k*. In particular, Poisson(*λ*) denotes the Poisson distribution with event rate *λ*. We simulated 500 samples of the following six point processes, samples containing on average 200 datapoints. Figure 3 displays example realizations from each class.

**Poisson**: We first sampled number of events *N*, where *N*∼Poisson(*λ*). We then sampled *N* points from a uniform distribution defined on the unit square [0,1]×[0,1]. Here *λ*=400.

**Normal**: Again a number of events *N* was sampled from Poisson(*λ*),*λ*=400. We then created *N* coordinate pairs (*x*,*y*), where both *x* and *y* are sampled from a normal distribution *N*(*μ*,*σ*^2^) with mean *μ* and standard deviation *σ*. Here *μ*=0.5 and *σ*=0.2.

**Matern**: A Poisson process as above were simulated with event rate *κ*. Obtained points represent parent points, or cluster centers, on the unit square. For each parent, a number of child points *N* was sampled from Poisson(*μ*). A disk of radius *r* centered on each parent point was defined. Then, for each parent the corresponding number of child points *N* were placed on the disk. Child points were uniformly distributed on the disks. Note that parent points are not part of the actual data set. We set *κ*=80, *μ*=5 and *r*=0.1.

**Thomas**: A Thomas process is similar to A Matern process except that instead of uniform distributions, child points were sampled from bivariate normal distributions defined on the disks. The distributions were centered on the parents and had diagonal covariance diag(*σ*^2^,*σ*^2^). Here *σ*=0.1.

**Baddeley-Silverman**: For this process, the unit square was divided into equal size tiles with side lengths $\frac {1}{28}$. Then for each tile number of points *N* was sampled, *N*∼Baddeley-Silverman. The Baddeley-Silverman distribution is a discrete distribution defined on values (0,1,10) with probabilities $(\frac {1}{10},\frac {8}{9},\frac {1}{90})$. For each tile, associated number of points *N* were then uniformly distributed on the tile.

**Iterated function system (IFS)**: We also generated point sets with an iterated function system. For this, a discrete distribution is defined on values (0,1,2,3,4) with corresponding probabilities $\left (\frac {1}{3},\frac {1}{6},\frac {1}{6},\frac {1}{6},\frac {1}{6}\right)$. We denote this distribution by IFS. A number of points *N* was then sampled, *N*∼Poisson(*λ*),*λ*=400. Starting from an initial point (*x*_0_,*y*_0_) on the unit square, *N* new points are generated by the recursive formula (*x*_*n*_,*y*_*n*_)=*f*_*i*_(*x*_*n*−1_,*y*_*n*−1_), where *n*∈{1,...,*N*},*i*∼IFS and the functions *f*_*i*_ are given as

$$f_{0}(y,x) = \left(\frac{x}{2},\frac{y}{2}\right), f_{1}(y,x)= \left(\frac{x}{2}+\frac{1}{2},\frac{y}{2}\right), f_{2}(y,x) = \left(\frac{x}{2},\frac{y}{2}+\frac{1}{2}\right)$$

$$f_{3}(y,x) = \left(\left|\frac{x}{2}-1\right|,\frac{y}{2}\right), f_{4}(y,x)= \left(\frac{x}{2},\left|\frac{y}{2}-1\right|\right). $$

### Tree branch data

**Fig. 4:**
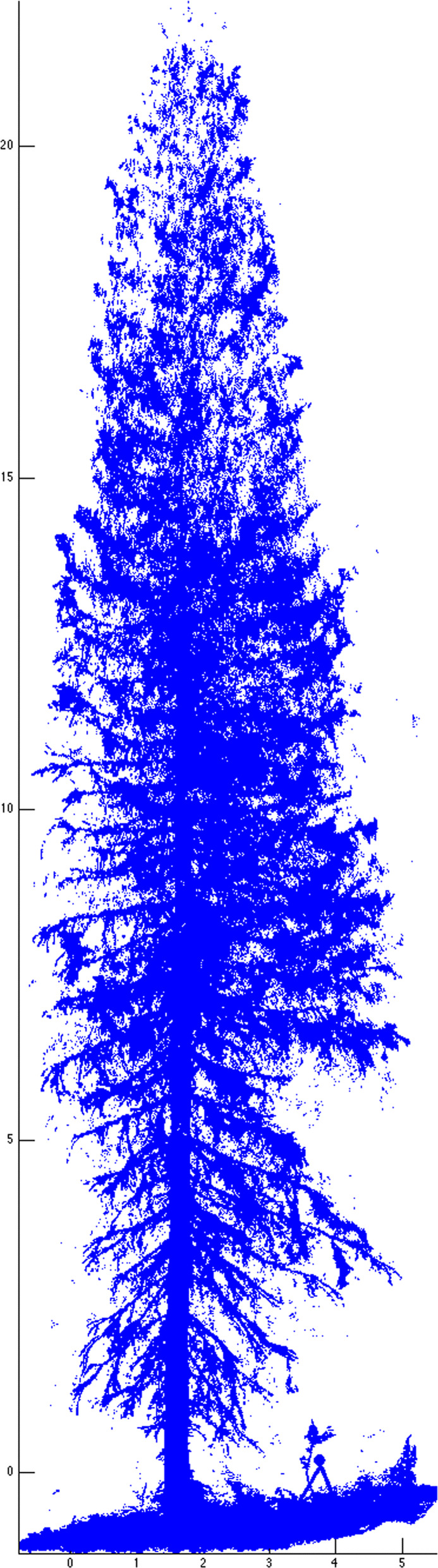
Example of laser scanned spruce point cloud. Courtesy of Raisa Mäkipää, see Acknowledgements

**Fig. 5:**
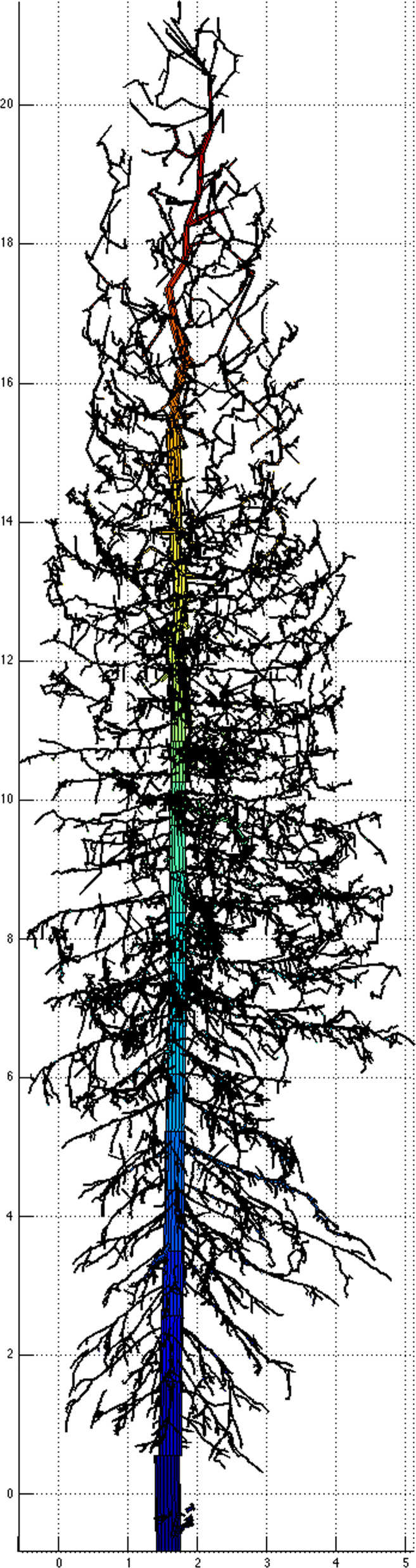
Example of QSM reconstructed model of the spruce point cloud in Fig. 5. Courtesy of Pasi Raumonen, see Acknowledgements

**Fig. 6:**
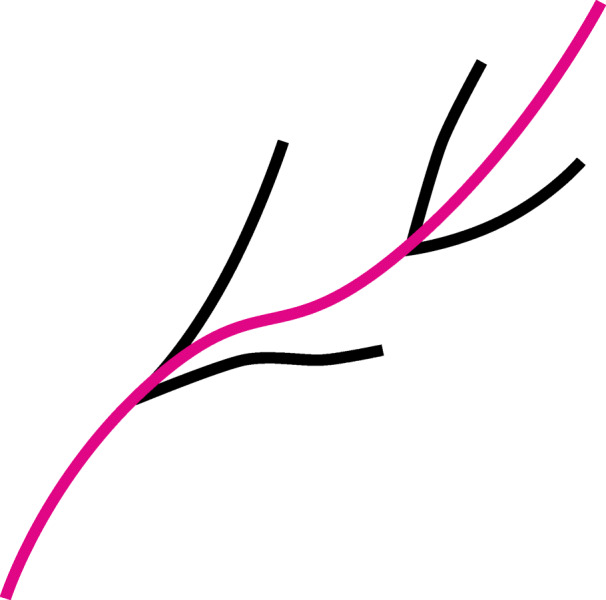
The purple main stem is the branch in our datasets, and is the parent branch of the black child branches

The second dataset came from Terrestrial Laser Scanning (TLS) of different tree species, representing a classification problem to correctly assign tree species from collected data. In this study we used data from Silver birch, Scots pine and Norway spruce. The scans were made in the location of Punkaharju in Finland. TLS produces point clouds of tree surfaces in 3D space. These point clouds can contain tens of millions of points and are not very useful for analysing tree data. A method of Quantitative Structural Modelling (QSM) for reconstructing tree models from TLS scans was developed in [15]. This method reconstructs trees by fitting cylinders in the point clouds. Figures 4 and 5 show, respectively, examples of laser scanned point cloud of a Finnish spruce and its QSM reconstruction. Reconstructed models make it possible to obtain diverse data from trees. For example, lengths and volumes of individual branches are obtained by summing the lengths and volumes of the cylinders making up the branch. QSMs also contain the topological structure of trees as parent-child relations between branches. For us, a branch means only the main stem excluding the child branches as shown in Fig. 6.

Tree structures are ubiquitous in biological organisms. Some recent studies have applied topological data analysis methods on brain arteries [9] and neurons [16]. Biological tree structures are very naturally modelled as tree graphs in 3D space [17, 18]. This, however, restricts the possibilities to obtain various data from the tree. Our approach is to view trees as point clouds of data and apply our topological analysis methods. As a multiple measurement case, we take one data point in a tree data set to be a branch of the tree with different features extracted from the QSM model. Specifically, branch datapoints had the features {branch order (0 for trunk, 1 for branches originating from trunk etc.), branch length in meters, branch height above ground in meters, angle between branch and upward z-direction in radians}. The trunk of the tree was excluded from the branch data. We had 100 samples from each tree species class, with each sample containing variable number of datapoints.

### Neuron spiking activity

The Blue Brain project aims to understand the relationship between the structure of the brain’s neuronal network and the observed activity of this network. To this end, the project has reconstructed a biologically realistic brain network, or neuronal microcircuitry [19]; more specifically a small region of the rat somatosensory cortex with 31,346 neurons. The reconstructed neurons have well-established morphological types and electrical behaviour inferred from in vitro brain slices. Structurally, the neurons are placed inside a small 3D volume according to experimentally based estimates of neuron densities inside the cortex and the synaptic connectivity between the neurons is reconstructed with models validated against observed anatomical data.

The reconstruction allows to simulate neurons’ electrical activity after injecting the network with an input signal. Electrical activity is measured as spiking of neurons, i.e. whether neuron releases built up electric potential, hence transmitting signal to connected neurons. We used simulation dataset from the study in [20]. In this study input signals were configured into nine different spatio-temporal patterns and injected into the reconstructed microcircuit. These stimuli differed mainly in the degree of spatio-temporal synchronizaton received by the input neurons.

The neuron dynamics were recorded in a spiketrain. We consider a spiketrain as a vector of 0’s and 1’s, where each element corresponds to a state of the neuron in time. A vector element with value 1 means that a neuron spikes at a time associated to the element, value 0 denotes no spiking activity.

Instead of looking at the spiketrains of individual neurons, we looked at spiking rates of communities of neurons. The neuronal network is a directed graph and community detection is major theme in network science [21]. We used the reverse idea and instead of finding communities, we selected communities with some criteria. A neuron community consists of a center neuron and neurons adjacent to this center. We choose 500 neuron communities from the full neuronal network around neurons having the highest degree (=number of incoming edges + number of outgoing edges). The community spiking rate is defined to be the number of neurons in the community spiking inside a specified time bin normalized by the number of neurons in the community. One 250 milliseconds brain activity simulation was therefore turned into a point cloud of 500 points in $\mathbb {R}^{250}$, where the vectors’ elements are community spiking rates in every 1 millisecond time bin. The nine different input signals corresponds to nine different simulation experiments labelled by *nXgY*, where *n* is in {5, 15, 30} labelling the temporal pattern and *g* is in {0, 1, 2} labelling the spatial pattern of the input signal. Each simulation was repeated 10 times with some randomized initial conditions. Altogether, in our nomenclature, we thus had 90 samples of neuron activity data, where each sample is a point cloud containing 500 datapoints.

## Results

**Fig. 7:**
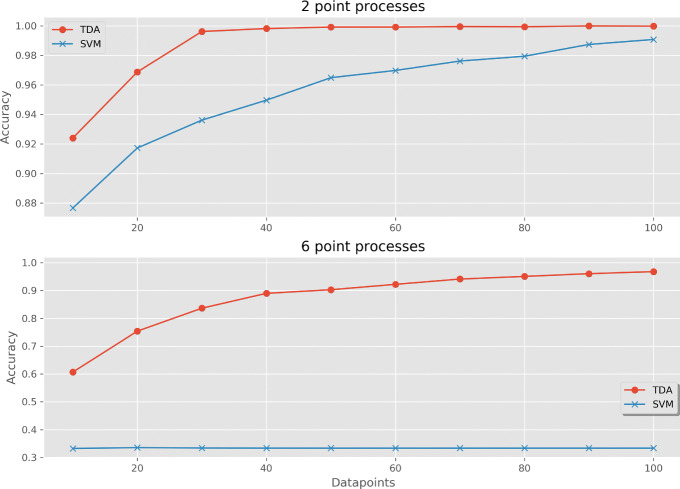
Cross-validated prediction accuracy when using a multi-measurement TDA classifier and compared with an SVM voting classifier. Datasets used were 2 classes of point processes (above) and 6 classes of point processes (below). Datapoints are the number of points sampled per point process realization

The first set of analyses was to test the algorithm on the point process and tree data. This was set up as a classification problem, wherein input data was used to predict the label. In both examples, the filter function used was the coordinate of the datapoint along the first principle component of the entire dataset, relative to the center of mass, and the metric was Euclidean distance. The linkage selected for local clustering was complete linkage. For point processes data, runs testing the TDA model as well as the alternative SVM model were conducted for sampling rates up to 100 datapoints from the list of sampling rates above, for both the full six point processes as well as for only normal and poisson point processes. The reason for the latter test was that the SVM appeared to have difficulty with the six class problem. The cross-validated accuracies are reported in Fig. 7.

Using six point processes, the TDA accuracy was 60.7% using 10 datapoints, and increased gradually to 96.8% using 100 datapoints. The alternative SVM model began with 33.2% using 10 datapoints, and remained at 33.3% during sampling to 100 datapoints.

Using only the two selected point processes, the TDA classifier achieved an accuracy of 99.6% after 30 datapoints, increasing to 99.98% at 100 datapoints of sampling. The alternative SVM model achieved an accuracy of 99.1% with 100 datapoints.

**Fig. 8:**
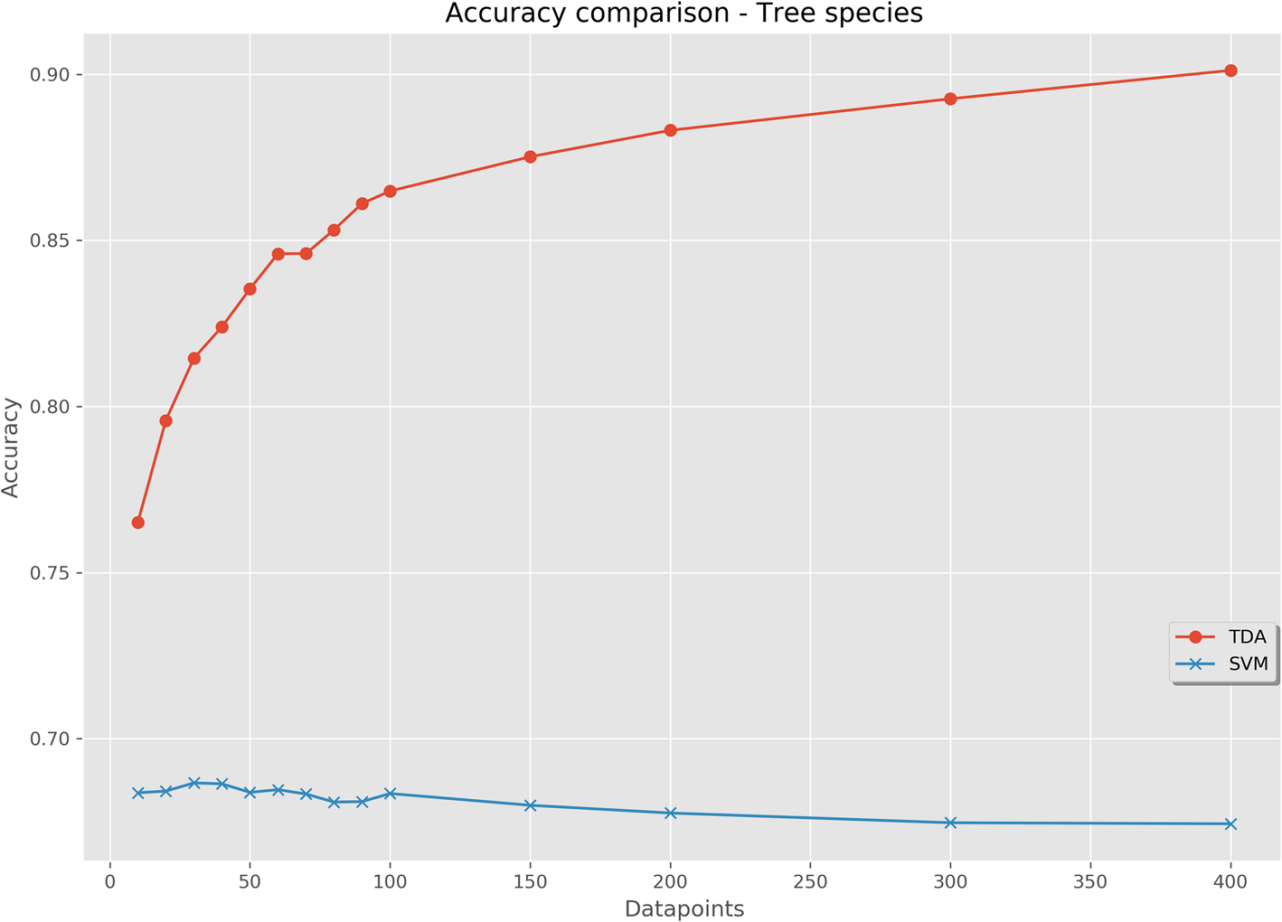
Cross-validated prediction accuracy when using a multi-measurement TDA classifier and compared with an SVM voting classifier. The dataset used was tree species, containing 100 samples in 3 classes. Datapoints are the number of points sampled per individual tree

The results of cross-validation for tree species is shown in Fig. 8. The TDA classifier had an accuracy of 76.5% using 10 sampled datapoints, increasing in an asymptotic manner until 400 sampled datapoints and an accuracy of 90.1%. The alternative model had an accuracy of 68.4%, increasing to a maximum of 68.7% using 30 datapoints, thereafter reducing slightly.

**Fig. 9:**
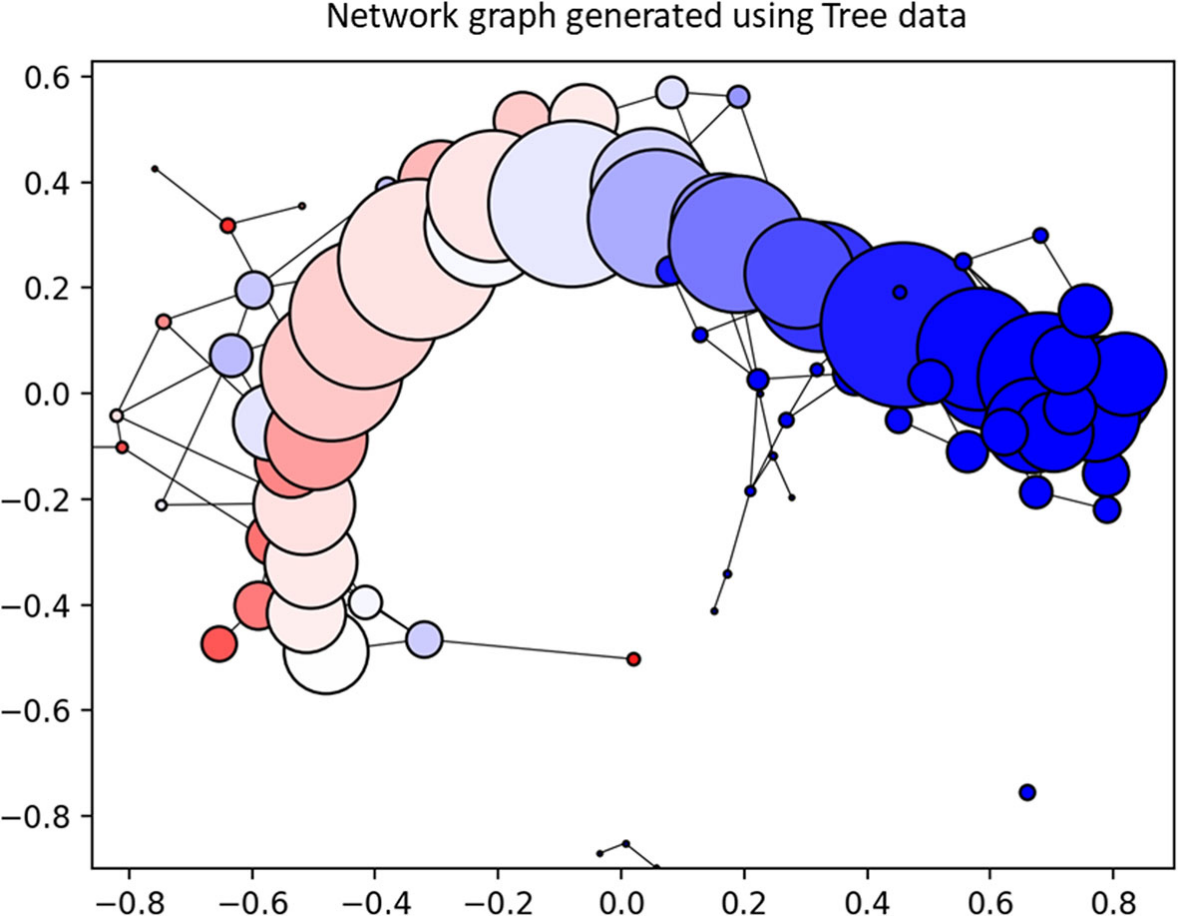
Graph generated using tree data of 100 samples in 3 classes. Each node represents a cluster of branches which co-aggregate in geometric space. The size of nodes are proportional to the number of branches, and the color represents the class proportion where darker blue color indicates a stronger proportion of one class and progressively more red indicates less pure nodes. The axis are in arbitrary units and the graph is not anchored

The next analysis of the tree data used node output generated from the software. A network graph visualizing the data and nodes created is shown in Fig. 9. Table 1 presents the top nodes in the full data model using 400 datapoints, ordered by number of datapoints. The purity, i.e. largest class proportion, gives important information about the suitability of each node as a tool for subgrouping data into unique partitions. The additional columns signify the average values of each feature for the given node. Similarly, this data can be used to find nodes with certain characteristics which are present in the data which inform the class membership. Ideally, this data can be used on the original dataset to better understand data partitioning and subclusters of various classes.

**Fig. 10:**
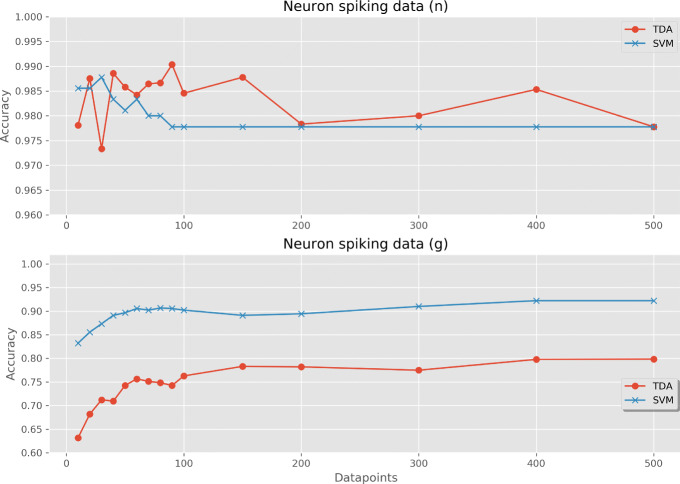
Cross-validated prediction accuracy when using a multi-measurement TDA classifier and compared with an SVM voting classifier. The datasets used were neuron spiking data with two class labelings, the temporal pattern *n* and the spatial-temporal pattern *g*

The final analysis of neuron spiking data gave differing results for classifying outputs related to temporal patterns *n* and spacial patterns *g*. Under *n*, the samples are divided into three classes. Under *g*, the samples are divided into nine classes corresponding to nine different *n*- *g* combinations. For temporal pattern classification, both methods resulted in high classification accuracies with even small numbers of datapoints sampled. The TDA classifier slightly outperformed the SVM in general, with both converging to 97.8% accuracy at 500 datapoints. For spatial pattern classification, the TDA classifier obtained 63.2% accuracy using 10 datapoints, compared with 83.2% for the SVM. This increase in accuracy for the SVM continued over all samplings, as shown in Fig. 10, with accuracies for the classifiers at 500 datapoints being 79.8% and 92.2% for the TDA and SVM methods, respectively.

## Discussion

This paper presents a method to analyze data featuring repeated measurements, in order to obtain high classification accuracy as well as information regarding features in the data which are important for the outcome of interest. In biological data, repeated measurements are often obtained, for example when sampling the same individual over time, when large amounts of data are sampled at a high frequency, or when blood is sampled and data from cell populations are obtained. This algorithm builds on the Mapper algorithm and extends it into the realm of machine learning.

The sampling procedure demonstrates that often only a relatively small number of datapoints is required to adequately model the data space in question in order to get high classification accuracy. Our method is both highly accurate on these datasets when compared with other methods, and importantly is able to determine which nodes are most responsible for the accuracy of the final model, such that determinations about complex relationships in the data can be extracted.

When using the algorithm, there are a few caveats that need to be taken into consideration. First, the user must avoid overfitting which could occur if testing a large number of filter functions and/or metrics, and thereafter selecting the best resultant model. Second, the results can be computationally intensive when the number of points sampled is high, due to runtime scaling to the order of *n*^2^. This can be remedied partially by reducing the size of the intervals in the underlying algorithm, which could be automatically scaled for larger datasets. Lastly, since this algorithm uses internal cross-validation the accuracy reported is based on a number of submodels which is equal to the number of cross-validation intervals. The final model which determines node characteristics includes all samples, therefore this internal accuracy may differ slightly from that of the unbiased estimate provided by the cross-validated accuracy.

The cross-validated accuracy of the TDA based classifier exceeds the alternative SVM voting classifier in most tests and sampling rates presented. This was consistent despite using only a single metric and filter function for the TDA model, while selecting the best kernel for the SVM based on accuracy. However, for determining the spatial pattern *g* label in neuronal spiking data, the SVM voting classifier exhibited a systematic increase in accuracy over the TDA model at all samplings. This clearly demonstrates that for some use cases, the TDA classifier does not provide an increase in accuracy over alternative methods, possibly due to inherent geometry that was not well suited for the filter function or clustering method used. These results might be improved by improved selection of parameters of the TDA model, if the caveats regarding overfitting are observed.

An interesting note about classification accuracies is that with an increased number of classes, the presented algorithm maintained a high accuracy when all point processes were used, but was outperformed for neuron spiking spatial patterns *g*. For six point processes, the alternate SVM classifier appears to maintain accuracy with two of the classes, while confusing the other four classes in a consistent basis, leading to the nearly constant 33% cross-validation accuracy. This surprising phenomenon possibly reflects a large variation in the data which does not lead to data organization which is accurately partitioned by a hyperplane. Similarly, a potential explanation for the TDA classifier’s high accuracy with more point process classes is that differences in point clouds which overlap in multi-dimensional space could require tools to tease out clusters based on similar geometry. Likewise, the results of the neuron spiking spatial patterns *g* indicate that geometric tools either may not be ideal when the underlying geometry of the data is not well suited for TDA or may require optimization of parameters for different use cases.

## Conclusions

The utility of this algorithm and implementation has broad applicability across the biological sciences as well as other fields. In particular, methods for obtaining repeated measurements classification models have been lacking, and our method fills a void in this manner. Furthermore, the ability to both partition data into its most useful components, and thereafter extract the features relevant for this partitioning, might allow researchers to identify which characteristics or variables in the data are most correlated with the outcomes.

Our algorithm and software can be employed by those who have repeated measurements data, and further extensions to this method can also be made. The application of topological data analysis demonstrates a scenario wherein data geometry becomes useful, and depending on the data characteristics, different metrics and filter functions can be applied. This demonstration of data analysis within the framework of machine learning and classification algorithms represents a novel utilization of TDA for common needs.

Additional development of methods using topological data analysis might result in further advances in classification techniques, and when combined with machine learning, there is strong potential for these methods in the future.

## Abbreviations

TDA: Topological Data Analysis
SVM: Support Vector Machine
RNA: Ribonucleic acid
T2D: Type-2 Diabetes
3D: Three dimension
PD: Probability Distribution
RBF: Radial Basis Function
IFS: Iterated function system
TLS: Terrestrial Laser Scanning
QSM: Quantitative Structural Modelling
